# SARS-CoV-2 in Urine May Predict a Severe Evolution of COVID-19

**DOI:** 10.3390/jcm10184061

**Published:** 2021-09-08

**Authors:** Alessandro Perrella, Mario Brita, Francesco Coletta, Simona Cotena, GiamPaola De Marco, Adele Longobardi, Crescenzo Sala, Dania Sannino, Antonio Tomasello, Marco Perrella, Giuseppe Russo, Marina Tarsitano, Massimo Chetta, Matteo Della Monica, Valentina Orlando, Enrico Coscioni, Romolo Villani

**Affiliations:** 1 Infectious Disease Service—Public Health Direction AORN A Cardarelli, 80100 Naples, Italy; giuseppe.russo@aocardarelli.it; 2 COVID-19 Intensive Care Unit—AORN A Cardarelli, 80100 Naples, Italy; mario.brita@virgilio.it (M.B.); francescoletta@libero.it (F.C.); cotenasimona@gmail.com (S.C.); giampaolademarco29@gmail.com (G.D.M.); adelelongobardi@gmail.com (A.L.); crescenzosala@hotmail.it (C.S.); dania.sannino1658@gmail.com (D.S.); doctom@libero.it (A.T.); Marco.perrella@aocardarelli.it (M.P.); romolo.villani@aocardarelli.it (R.V.); 3 Molecular Biology and Clinical Genetic Unit, 80100 Naples, Italy; marina.tarsitano@aocardarelli.it (M.T.); Massimo.chetta@aocardarelli.it (M.C.); matteo.dellamonica@aocardarelli.it (M.D.M.); 4 CIRFF, Center of Drug Utilisation and Pharmacoeconomics, University of Naples Federico II, 80100 Naples, Italy; valentina.orlando@unina.it; 5 Division of Cardiac Surgery, AOU San Giovanni di Dio E Ruggi d’Aragona, 84100 Salerno, Italy; Enricocoscioni@gmail.com

**Keywords:** COVID-19, SARS-CoV-2, unusual COVID, atypical COVID-19, SARS-CoV-2 urine

## Abstract

We hypothesized that the spread of SARS-CoV-2 in urine during a severe COVID-19 infection may be the expression of the worsening disease evolution. Therefore, the aim of this study was to verify if the COVID-19 disease severity is related to the viral presence in urine samples. We evaluated the clinical evolution in acute COVID-19 patients admitted in the sub-intensive care and intensive care units between 28 of December 2020 and 15th of February 2021 and being positive for SARS-CoV-2 RNA in the respiratory tract, including repeated endotracheal aspirates (ETA), sputum, nasopharyngeal swabs (NPS) and urine. We found that those subjects with SARS-COV-2 in the urine at admittance (8 out of 60 eligible patients) had a more severe disease than those with negative SARS-CoV-2 in urine. Further, they showed an increase in fibrinogen and (C-reactive Protein) CRP serum levels, requiring mechanic ventilation. Of those with positive SARS-CoV-2 in the urine, 50% died. According to our preliminary results, it seems that the presence of SARS-CoV-2 in the urine characterizes patients with a more severe disease and is also related to a higher death rate.

## 1. Introduction

Since the very early phase of pandemic, the SARS-CoV-2 infection (COVID-19) showed to be a serious challenge to public health and, while persisting, continues to underline the emergency it represents [1]. The mainly clinical feature of this virus is related to respiratory function; however, growing evidence has shown the involvement of several other organs. Therefore, symptoms may vary from mild/asymptomatic to critical, the latter being characterized by acute respiratory failure and multiorgan thrombotic events, according to different clinical presentations [1,2].

Many factors seem to influence the clinical evolution as well as the natural history of the disease, such as lymphocytopenia, or comorbidities, such as diabetes, heart failure or chronic disease, as well as variants of concern [3]. However, the pathogenesis of the infection and laboratory parameters that could be helpful to predict the disease evolution are still objects of research [4]. Throughout the last year, the importance of rt-PCR of SARS-CoV-2 on nasal swabs has been proposed, particularly regarding cycle threshold (Ct) evaluation in relation to contagiousness and infection [5,6]. In particular, SARS-CoV-2 detection and viral load at different time points of infection, including in those without any symptoms, would help to better understand the disease evolution [6,7]. Indeed, while rRT-PCR by a nasopharyngeal swab is a well-defined method for the diagnosis of COVID-19, little data exist to assess the role of positive rRT-PCR in other biologic samples and its possible correlation with the disease severity and infectiousness to others [8,9,10]. In fact, disease severity is triggered by several predisposing factors, including viral shedding, that could be related to the immune response and infection control [11,12]. Indeed, COVID-19, as previously suggested, which shows systemic involvement [13], is also characterized by endothelium damage with an increase in fibrinogen serum levels [14] and is correlated to a worse prognosis [14,15,16]. According to this evidence, we hypothesized that the inflammatory endothelial damage may determine viral shedding in urine and be an expression of a further worsening systemic dissemination of SARS-CoV-2, possibly with a worse outcome [17]. Indeed, the idea that viral shedding in the urine could be the expression of systemic involvement of the infection has been previously found in other viral disease and more recently in COVID-19 too [18]. Consequently, the aim of this study was to verify if COVID-19 disease severity in patients admitted to our hospital is related to the viral presence in urine samples with a systemic inflammatory response too.

## 2. Material and Methods

AORN A Cardarelli is a major Emergency Hospital of South Italy with more than 90,000 admissions per year. During the COVID-19 pandemic, at the beginning of February, more than 1300 COVID-19 patients were admitted in the sub-intensive and intensive care units. This observational study evaluated all hospitalized COVID-19 patients admitted to the Padiglione M COVID-19 Intensive Care Unit at AORN A Cardarelli Hospital between the 28th December 2020 and the 15th February 2020. Inclusion criteria were as follows: all patients should have a positive SARS-CoV-2 nasopharyngeal swab specimen collected within 24 h of admission. Subjects under the age of 18, current inpatients, those with initial SARS-CoV-2 testing > 48 h after admission, or missing BMI or with a previous history of COVID-19 were excluded from the analysis. On the contrary, negative serology for SARS-CoV-2 and a cycle threshold (Ct) in the real-time polymerase chain reaction (RT-PCR) lower than 30 was considered as the expression of an acute SARS-CoV-2 infection [13]. All patients at the time of admission gave informed written consent according to Italian healthcare policy and were treated according to the Helsinki declaration [14]. The patient information and data, including test results and comorbidities, were collected retrospectively from electronic medical records. Vital sign data included in the study represented the first recorded vitals in the hospital during admission to the emergency room, and the biochemical and other parameters were indexed as the closest result within 48 h of SARS-CoV-2 testing. Data about previous COVID-19 infection statuses were evaluated according to the centralized Campania Region DB called Sinfonia https://sinfonia.soresa.it/sinfonia/ (accessed on 28 December 2020). COVID-19 was diagnosed with evidence of SARS-CoV-2 RNA in material from the respiratory tract, including repeated endotracheal aspirates (ETA), sputum and nasopharyngeal swabs (NPS), at admission. Urine samples were collected at the T0 too and RT-PCR was performed as described below. These samples were repeated during the hospital stay according to the clinical condition (improvement or worsening) or based on infectious disease specialist evaluation. At least every patient received 4 COVID-19 PCR tests during his/her hospital stay. Concerning RT-PCR in urine and respiratory samples, viral RNA was extracted from 300 µL of urine and 300 µL of Naso-pharyngeal swab samples with an automated extraction instrument according to the manufacturer’s protocol (TanBead, Taiwan Advanced Nanotech Inc., Taiwan,) and previous scientific evidence (results as described previously) [12]. SARS-CoV-2 were detected by a real-time reverse transcription polymerase chain reaction (RT-PCR) assay using the Allplex™ SARS-CoV-2 Assay kit according to the manufacturer’s protocol (Seegene Inc., Seoul, Korea). The assay is designed to detect RdRP, S and N genes specific to SARS-CoV-2, and the E gene during the real-time RT-PCR assay. Repeated collection of either sample (NPS, sputum, and ETA) was performed for clinical monitoring. When COVID-19 symptoms receded and two consecutive NPS (at least with a day distance) showed a negative result, testing was stopped. COVID-19 resolution was defined as clinical when all clinical signs of SARS-CoV-2 infection were found to be resolved at least 3 days, while complete disease resolution was defined according to two negative RT-PCR samples.

Clinical condition was assessed according to a simple classification for disease severity: severe cases were defined as patients with the need for mechanical ventilation, as it was used before [15]. Moderate disease in our patients was defined by the absence of mechanical ventilation and the need for oxygen insufflation, while the absence of both defined mild disease courses. Non-severe disease includes mild to moderate disease according to our experiences based on the clinical score known as the “Cardarelli Score” (Table 1) and previous evidence [15] to assess our scoring system too. All patients underwent regular clinical, laboratory and imaging follow-ups, as shown in Table 2. Vital parameters, such as respiratory frequency, O_2_ saturation and the PaO_2_/FiO_2_ ratio, were also collected as well as Arterial blood gas (ABG). In addition, all patients were followed during the observation period on their clinical evolution. In particular, we used CRP, IL-6 and fibrinogen serum levels as inflammation and endothelial damage marks, as assessed in our centralized laboratory. Renal and liver function was performed routinary during the hospital stay, and MDRD 6 variables, CHILD and MELD scores were performed. When kidney or liver function was found to be altered, according to Hospital protocols, specialist consulting were performed. Statistical analysis to assess possible differences was performed according to non-parametric Tests (Mann-Whitney U two-tailed) and were evaluated on GraphPad and Jamovi 4.0 for MacOS.

## 3. Results

In the last three months of 2020, the Campania Region faced a second wave of the COVID-19 pandemic. Throughout this time, we observed an increase in patient admission being characterized by more significant disease severity with higher inflammation markers, such as as (C reactive protein) CRP, Interleukin 6 (IL-6), Fibrinogen and altered Neutrophil/Lymphocyets (NLR) ratio, with the worst prognosis in elder people than previously experienced during the first phase of the pandemic in March 2020. Here, we report the clinical and virological findings describing the dynamic of SARS-CoV-2 viral shedding in a cohort of 85 consecutive patients admitted to our hospital between 28 December 2020 and 15 February 2020. In particular, we found 75 out 85 patients were eligible according to our criteria with a mean hospital stay of 18 ± 4 days. Clinical features at the admission were as follows: fever (63%), dyspnea (45%), Lymphopenia (99%), elevated neutrophil-to-lymphocyte ratio(NLR) (97%), elevated levels of CRP on the laboratory cut-off (98%), an increase in lactatedehydrogenase (LDH) (77%), higher ferritin (65%) and an increase in d-dimer (87%) and fibrinogen (82%), according to previous evidence on COVID-19 [13,16,17] (Table 2). However, according to clinical classification in Table 1, we found that: the fibrinogen serum level was higher in severe vs. moderate diseases (*p* > 0.05 Mann-Whitney U Test two-tailed, with *z*-score −3.69231, and the *U*-value is 12. The critical value of *U* at *p* < 0.05 is 45). Further, the IL-6 serum level was increased in severe vs. moderate diseases (*p* > 0.05 Mann-Whitney U Test two-tailed, with *z*-score −4.17949, and the *U*-value is 2.5. The critical value of *U* at *p* < 0.05 is 45). At the presentation of the disease, 60 out of 75 patients had Ct values lower than 30 in the nasal swab with negative antibodies anti-SARS-CoV-2 and were considered in the acute phase of infection [8]. Out of 75 subjects, 15 were patients with Ct > 30 but lower than 35 and had a history of infection for more than 7 days with clinical worsening and were therefore excluded. Out of 60 patients with an acute infection, 25 had a severe disease, 18 patients had a moderate disease, while 17 had a mild disease according to the classification we used [15] and our internal scoring system. Patients with a mild disease only needed a small amount of oxygen on admission and during their hospital stay, while those with a moderate or severe disease showed a change in oxygen demand. Specifically, 15 out 25 patients with a severe disease upon admission required mechanical ventilation. Out of 60 patients, 15 had a fast negative PCR within 6 days from presentation, having almost absent inflammation markers. Of those subjects, 14 patients were from those with a mild disease and 1 from the moderate disease group. Interestingly, all these patients showed a higher lymphocytes cell count compared to those with a longer disease course (800 cells/mmc vs. 400 cells/mmc, lower limit n.v. 1000 cells/mmc; (*p* > 0.05 Mann-Whitney Test U two-tailed, with *z*-score 4.80534, and the *U*-value is 0. The critical value of *U* at *p* < 0.05 is 75). No positive SARS-CoV-2 RT-PCR was found when urine was tested upon admission in the patients with fast viral clearance. Concerning the 50 remaining patients, 7 out 50 had SARS-CoV-2 positive rt-PCR in urine at the time of the admission. Six out seven of those patients had a four gene positive PCR, and just one had only RdP gene positive at the RT-PCR assay (Table 2). Every patient had ground-glass opacity, and half of the patients had a consolidation on the CT scan with a mean score of 16 out 24 according to disease severity [19]. We did not experience any worsening of liver or renal function in our patients. Moreover, renal function did not show any alteration in both those with positive and negative SARS-CoV-2 in the urine. We only have three patients presenting oliguric syndrome in those without evidence of SARS-CoV-2 in urine.

**Figure 1:**
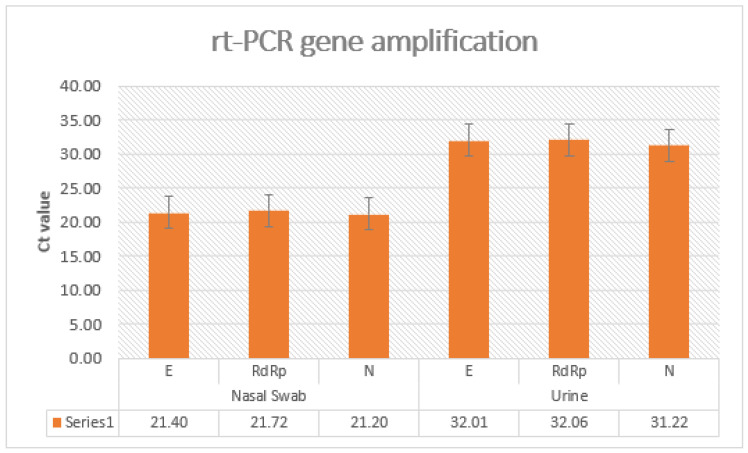
The Ct value at 3 genes analysis with a range value for the cycle threshold of patients with positive SARS-CoV-2 PCR in a nasal swab and urine.

The CT scan score of those 6 patients with positive urine was 21 out of 24 (Table 2). Of the 15 patients with respiratory worsening, six were those having positive urine for SARS-CoV-2. Further, the patients with viral shedding in urine had higher CRP and fibrinogen than those with negative SARS-CoV-2 in urine (Figure 1). However, due to the small number of subjects, an analysis to evaluate statistically significant differences cannot be done; therefore, we only observed this increase in CRP and fibrinogen. Unfortunately, we had four deaths, of whom three were in those subjects with viral shedding in urine.

## 4. Discussion

Previously, SARS-CoV-2 viral shedding has been found in stool samples, suggesting a possible role in those with gastrointestinal symptoms; however, clinical significance when found in other biological samples still remains unclear [18]. In our observational study on 60 high viremic patients with acute COVID-19, we found that seven patients (11.66%) not only had viral shedding in urine but also presented higher inflammation markers, such as CRP, IL-6 as well as Fibrinogen. Further, the above-mentioned patients needed intensive care oxygen support, and three out of those seven patients died, still showing positive rtPCR in their urine and respiratory samples (Table 1). On the contrary, the remaining patients showing positive SARS-CoV-2 PCR in urine, after intensive care support, required three weeks of further hospital stay and therefore were dismissed in the ordinary ward as being SARS-CoV-2 negative. Another interesting finding is related to the N/L ratio and lymphocytes cell count being lower in those with positive urine for SARS-CoV-2. The reason for the lower count in lymphocytes and therefore in the N/L ratio may account for a more efficient immune response.

Indeed, while RT-PCR by a nasopharyngeal swab is effective for the diagnosis of COVID-19, little data exist to assess if it could correctly reflect the systemic disease severity and infectiousness to others and/or the expression of the ability of the immune system to contain a viral spread. Indeed, the relationship between SARS-CoV-2 detection, viral load and infectivity is not fully understood, as the presence of viral RNA may not represent transmissible live virus or the expression of the immune system’s defective response [7,8,9,10]. Even though our findings are on a small cohort of patients, they clearly suggest that a viral shedding in urine during natural history of disease is characterized by a worsening outcome of the patients regardless of organ function. Thus, this finding could be an expression of an inflammation state of endothelium, as higher serum levels of fibrinogen and IL-6 seem to suggest and as previously suggested [16,17,18,20]. Of note, previously, inflammatory markers were found to be related to a severe endothelial injury with cellular death/apoptosis, and the presence of intracellular virus in the autopsy lung with thrombosis, and small- to middle-sized pulmonary vessels were considered as an expression therefore of an increase in disease severity [13,14,15]. The evidence of positive RT-PCR in urine with a hyper-inflammatory state with endothelial damage engagement would suggest that once a systemic phase has been reached with kidney involvement in terms of viral shedding, the disease could have a worse prognosis, as occurs for the septic state [21]. Certainly, the evidence that a systemic viral shedding, particularly in the urine, could represent a marker of disease severity and a worse prognosis could be carefully used. In fact, it would be an interesting strategy to also assess with COVID-19 to determine a prompt hospital admission with specific therapeutic strategies aiming to reduce the inflammatory status and endothelial damage. Interestingly, according to previous evidence too, the presence of the virus in the urine did not influence renal function [22]. This could be associated with the fact that viral RNA may pass through kidneys due to vascular permeability related to the systemic inflammation environment, as also occurs in the Dengue virus [23]. Even though our paper is based on a small sample, it shows for the first time that viral shedding in urine may be associated with a worse outcome and, therefore, input for future studies. In conclusion, viral shedding of SARS-CoV-2 in the urine at admission to the hospital and its persistence during hospitalization could be an expression of more severe disease, and it seems to be correlated to a worse clinical course. However, further studies are required on a larger population to better assess this hypothesis and possible future implications.

## Figures and Tables

**Table 1:** Cardarelli Hospital COVID-19 Clinical scoring System.

	Scoring System		
	Score 1	Score 2	Score 3
Lymphocytes (>1000 cell/mmc)	601–1000	401–600	<400
CRP (<5 mg)	>10 x n.v.	>20 x n.v.	<25 x n.v.
P/F ratio	≥250	250–150	<150
CT Scan Score *	<10/25	11–18/25	>18/25
Fever for more than 3 days	<38 °C	38–39 °C	>39 °C
Dyspnea	mild	moderate	severe

Table 1 shows the scoring system of patients admitted in sub-intensive or intensive care units. A score of 6–10 points was mild disease, 12–14 points was moderate disease, while 18 was considered severe disease. Further patients having 2 Score 2 or Score 3 parameters were considered, respectively, with mild or severe disease. * [19].

**Table 2:** Data show laboratory findings and parameters of the hospitalized patients with or without RT-PCR SARS-CoV-2 positive in urine. Data are expressed as the absolute number, percentage and S.D.

Variables	Total	Urine Negative (Acute Infection)	Urine Positive (Acute Infection)
	(*n* = 60)	(*n* = 53)	(*n* = 7)
**Demographic data**			
Age (years) Sex	68 ± 5	65 ± 6	67 ± 3
Male	16 (84%)	9 (75%)	7 (100%)
Female	3 (16%)	3 (25%)	0 (0%)
Smoking history			
Yes	13 (68%)	7 (58%)	6 (86%)
No	6 (32%)	5 (42%)	1 (14%)
**Comorbidities**			
Hypertension	12	4	2
Diabetes	6	3	1
Cardiovascular disease	8	4	3
Chronic liver disease	2	0	0
Chronic lung disease	4	2	0
Chronic kidney	3	0	0
disease Cancer	5	2	1
Immunocompromising conditions	3	1	0
**Signs and symptoms**			
Fever	12	8	4
Cough	9	6	3
Fatigue	8	3	5
Diarrhea	3	2	1
Shortness of breath	13	9	4
**Laboratory findings**	5.753 (±204)	6.355 (±159)	4.673 (±109)
WBC (cells/mmc)	1530 (±71)	996 (±52)	628 (±64)
Lymphocyte (cells/mmc)	4.0 (5.2)	4.3 (4.2)	3.2 (14.2)
NLR (Neutropylete/lymphocyte ratio)	88.1	72.1	85
CRP (mg/L)	25 (±15)	24 (±11)	35 (±12)
AST (U/L < 50)	267 (±43)	312 (±15)	318 (±29)
ALT (U/L < 40)	273 (±159)	300 (±192)	234 (±711)
LDH (U/L < 250)	602 (±117)	425 (±231)	835 (±194)
Fibrinogen (ng/mL)	2.6 (4.9)	3.2 (7.6)	1.8 (3.4)
D-dimer (ng/mL)	257 (12.1)	28.4 (9.4)	22.2 (5.4)
Creatinine Clearance (MDRD 6 variable)	62 ± 2 mL/min	61 ± 2 mL/min	58 ± 3 mL/min
**Ct 4 gens mean value of RT-PCR**	20.8	18.2	21.3
Respiratory Function	25 (±15)	24 (±11)	35 (±12)
P/F Ratio	172 ± 14	170 ± 12	145 ± 22
NIV (Non-Invasive ventilation) (as %)	44%	45%	43%
Venturi Mask (as %)	32%	40%	0%
Intubation (as %)	24%	15%	57%
CT scan findings			
T Score Wang (x/24)	19/24 (±1)	18/254 (±1)	21/24 (±2)

## Data Availability

No public repository.
